# Potential of Endocannabinoids to Control Bladder Pain

**DOI:** 10.3389/fnsys.2018.00017

**Published:** 2018-05-15

**Authors:** Dale E. Bjorling, Zun-yi Wang

**Affiliations:** School of Veterinary Medicine, University of Wisconsin-Madison, Madison, WI, United States

**Keywords:** bladder pain, visceral pain, endocannabinoids, 2-arachidonoylglycerol, anandamide, fatty acid amide hydrolase

## Abstract

Bladder-related pain is one of the most common forms of visceral pain, and visceral pain is among the most common complaints for which patients seek physician consultation. Despite extensive studies of visceral innervation and treatment of visceral pain, opioids remain a mainstay for management of bladder pain. Side effects associated with opioid therapy can profoundly diminish quality of life, and improved options for treatment of bladder pain remain a high priority. Endocannabinoids, primarily anandamide (AEA) and 2-arachidonoylglycerol (2-AG), are endogenously-produced fatty acid ethanolamides with that induce analgesia. Animal experiments have demonstrated that inhibition of enzymes that degrade AEA or 2-AG have the potential to prevent development of visceral and somatic pain. Although experimental results in animal models have been promising, clinical application of this approach has proven difficult. In addition to fatty acid amide hydrolase (FAAH; degrades AEA) and monacylglycerol lipase (MAGL; degrades 2-AG), cyclooxygenase (COX) acts to metabolize endocannabinoids. Another potential limitation of this strategy is that AEA activates pro-nociceptive transient receptor potential vanilloid 1 (TRPV1) channels. Dual inhibitors of FAAH and TRPV1 or FAAH and COX have been synthesized and are currently undergoing preclinical testing for efficacy in providing analgesia. Local inhibition of FAAH or MAGL within the bladder may be viable options to reduce pain associated with cystitis with fewer systemic side effects, but this has not been explored. Further investigation is required before manipulation of the endocannabinoid system can be proven as an efficacious alternative for management of bladder pain.

## Introduction

Despite the exceedingly common occurrence of visceral pain, far less is known about the anatomy and physiology that underlie visceral pain relative to that associated with somatic pain. An excellent recent review of the physiology of visceral pain pointed out that afferent innervation of viscera consists of “either vagal and spinal nerves *or* two anatomically distinct sets of spinal nerves” (Gebhart and Bielefeldt, 2016). The diffuse and somewhat sparse nature of afferent visceral innervation results in poorly localized discomfort that is often perceived as pain referred to somatic structures, possibly as a result of cross-communication between afferent visceral and somatic nerves as they comingle in peripheral ganglia, dorsal root ganglia, the spinal cord, or higher centers (Pierau et al., 1984; Arendt-Nielsen et al., 2000; Craig, 2003; Farrell et al., 2014; Luz et al., 2015; Lovick, 2016).

Patients with visceral pain thought to arise from specific organs, such as the bladder or bowel are treated with a variety of analgesics, including opioids, but failure to respond, alterations in pain sensitivity, decreased bowel motility and addiction are unfortunately common in these patients (Quang-Cantagrel et al., 2000; Brock et al., 2012; Wang et al., 2017; Weber et al., 2017). Alternative therapies such as electrostimulation of nerves, immunotherapy and homeopathic remedies have been used with mixed, but typically poor or transient, results in these patients (Farhadi et al., 2001; John et al., 2003; Capodice et al., 2005; Brock et al., 2008; Mykoniatis et al., 2017).

Treatment of visceral pain thought to arise specifically from the bladder has included instillation of compounds into the bladder or distention of the bladder. The efficacy of various intravesical treatments was recently reviewed (Zhang et al., 2017). This report observed that botulinum toxin A, bacillus Calmette-Guerin, and pentosan polysulfate showed the greatest promise. Distention of the bladder provides transient relief in some patients, but the mechanism for this remains unknown. Data in support of the efficacy of this treatment are relatively weak, and duration of positive effects are relatively short-lived (Erickson et al., 2007; Hoke et al., 2017; Olson et al., 2018). Translation of experimental findings generated in rodent models of acute or chronic bladder inflammation to clinical practice has proven difficult in patients with persistent bladder pain of long duration.

Recent developments in legalization of cannabis or cannabinoid products has increased interest in these compounds as an alternative therapy for pain. Systemic administration of exogenous cannabinoids to control pain appears to be most efficacious in patients with cancer-related pain (Tateo, 2017). The capacity of cannabinoids to decrease nausea and pain in cancer patients has been described by multiple authors, albeit often in the presence of side effects relating to altered mentation (Johnson et al., 2010; Abrams and Guzman, 2015; Davis, 2016). A recent meta-analysis found that pre-clinical studies using animal models of pain strongly supported the capacity of cannabinoids to reduce opioid doses, but clinical trials to date have failed to support this observation (Nielsen et al., 2017). Similarly, a meta-analysis of studies revealed that relief of non-cancer pain by cannabinoids was extremely weak and accompanied by significant side effects in these patients (Allende-Salazar and Rada, 2017). Short-term adverse side effects of smoked cannabis include anxiety, agitation, illusions, feelings of depersonalization, hallucinations, paranoid ideation, temporal slowing, impaired judgment/attention, red eyes, dryness of the mouth, tachycardia and increased appetite (Zhang and Ho, 2015), and occasionally, hyperemesis and intestinal perforation (Buyukbese Sarsu, 2016; Dezieck et al., 2017). An alternative to management of bladder pain by administration of exogenous cannabinoids is manipulation of endocannabinoids.

## Endocannabinoid Metabolism

As the name implies, endocannabinoids are endogenously synthesized fatty acids with chemical structures similar to those of biologically active exogenous cannabinoids. Endocannabinoids are hydrophobic, neutral lipids that are not stored within cells but rather are produced on demand (Marsicano et al., 2003). Post-synaptic neurons rapidly synthesize endocannabinoids that bind to cannabinoid 1 (CB1) and 2 (CB2) receptors on presynaptic neurons to inhibit signaling; endocannabinoids are then metabolized by enzymes within presynaptic neurons (Piomelli et al., 1998; Alhouayek and Muccioli, 2012; Kohnz and Nomura, 2014). N-arachidonoyl ethanolamine (anandamide or AEA) and 2-arachidonoylglycerol (2-AG) are the most widely recognized and best characterized endocannabinoids, and 2-AG is the most abundant endocannabinoid identified to date (Palmer et al., 2002; Di Marzo et al., 2004; Kogan and Mechoulam, 2006). The presence and activity of endocannabinoids are tightly controlled by enzymatic degradation (Ahn et al., 2008); 2-AG is primarily degraded by monoacylglycerol lipase (MAGL) and AEA by fatty acid amide hydrolase (FAAH; Bisogno et al., 1997; Thomas et al., 1997; Goparaju et al., 1999). Strategies for testing management of visceral pain (and other types of pain) by endocannabinoids have primarily focused on inhibition of degradation of AEA or 2-AG by administration of compounds that inhibit the function of FAAH and MAGL or generation of mice lacking functional FAAH or MAGL.

Cyclooxygenase 1 and 2 (COX1 and COX2), but primarily COX2, also participate in degradation of AEA and 2-AG by oxygenation of these compounds to the corresponding PGH2 analogs (Kozak et al., 2000, 2001; Weber et al., 2004; Goodman et al., 2018). The primary function of COX is conversion of arachidonic acid to prostaglandins that promote inflammation and pain, and inhibition of COX has become standard first line therapy for acute inflammatory pain (Smith et al., 2000; Fleckenstein et al., 2016). However, COX antagonists alone do not adequately control visceral pain (Fleckenstein et al., 2016). Interestingly, it has been reported that nonsteroidal drugs that inhibit COX activity may also suppress the function of FAAH (Fowler et al., 1997, 1999), and intrathecal administration of COX inhibitors suppressed *in vivo* pain induced by subcutaneous formalin injection in the rat (Guhring et al., 2002; Ates et al., 2003), as well as release of calcitonin gene-related peptide (pro-algesic neuropeptide) in response to *in vitro* exposure of rat spinal cord to capsaicin (Seidel et al., 2003). The salient effects of COX antagonists in both studies were inhibited by administration of an antagonist to CB1. A dual inhibitor of FAAH and both isoforms of COX suppressed experimentally-induced gastrointestinal inflammation, as well as pain and edema due to subcutaneous injection of carrageenan in mice (Sasso et al., 2015). This compound is particularly interesting, because its chemical structure is such that it also has high efficacy in inhibition of degradation of 2-AG by COX (Goodman et al., 2018).

## Cannabinoid Receptors

Endocannabinoids inhibit nociception primarily by binding to G-protein coupled receptors, CB1 and CB2, in peripheral tissue, spinal cord and brain. CB1 and CB2 have also been identified in the bladders of mice, rats, monkey and humans, primarily within the urothelium and afferent and cholinergic nerves within the bladder wall (Hayn et al., 2008; Gratzke et al., 2009, 2010; Tyagi et al., 2009; Merriam et al., 2011; Bakali et al., 2013, 2016; Wang et al., 2013). The function of CB1 and CB1 located on nerves has been extensively investigated, but the functional purpose of expression of cannabinoid receptors by non-neuronal tissue remains largely unknown. A summary of studies reporting localization of cannabinoid receptors in the bladder has been published (Hedlund, 2014).

CB1 is particularly abundant in the brain and plays a significant role in modulation of nociceptive signaling in the central and peripheral nervous systems (Wilson and Nicoll, 2002; Agarwal et al., 2007; Lau et al., 2014). A majority of somatic and visceral afferent nerve fibers express CB1 (Hohmann and Herkenham, 1999; Ahluwalia et al., 2000; Agarwal et al., 2007), and genetic deletion of CB1 from afferent nerves renders mice sensitive to subthreshold stimuli (allodynia) and enhances response to noxious stimuli (hyperalgesia; Agarwal et al., 2007). We demonstrated that CB1 receptors have the capacity to suppress the sensitizing effect of nerve growth factor (NGF) on the response of mouse afferent neurons to capsaicin *in vitro* (Wang et al., 2015a). Interestingly, we also showed that intrathecal administration of the CB1 agonist arachidonyl-2’-chloroethylamide (ACEA) blocked referred mechanical hypersensitivity induced by inflammatory cystitis in rats (Jones et al., 2015).

CB2 receptors are present on immune cells, as well as within the brain, spinal cord and peripheral afferent nerves (Galiegue et al., 1995; Ibrahim et al., 2003; Wotherspoon et al., 2005; Anand et al., 2009; Graham et al., 2010). Multiple studies have demonstrated analgesic effects of activation of CB2 in neuropathic or inflammatory pain models (Ibrahim et al., 2003; Nackley et al., 2004; Gutierrez et al., 2007; Anand et al., 2008). We have previously reported inhibition of increased referred mechanical sensitivity by systemic treatment of mice with CB2 agonists administered prior to Wang et al. (2013) or after Wang et al. (2014) induction of bladder inflammation. Thus, our prior work has demonstrated the capacity of activation of CB1 and/or CB2 receptors to block increased peripheral mechanical sensitivity accompanying inflammatory cystitis, and further that this may in part be due to suppression of the sensitizing effects of NGF on afferent nerve signaling.

## Inhibition of FAAH

**Figure 1:**
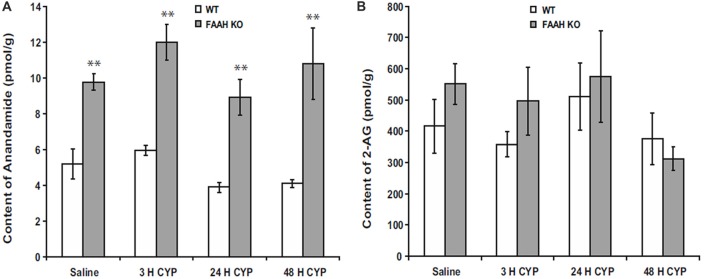
Bladder content of anandamide (AEA) **(A)** were consistently greater in fatty acid amide hydrolase (FAAH) knock out (KO) mice than wild-type (WT) mice treated with saline (controls) or cyclophosphamide (CYP; 150 mg/kg) given intraperitoneally 3 h prior to sacrifice. Bladder content of 2-arachidonoylglycerol (2-AG; **B**) was similar in both KO and WT and was unaffected by treatment. Mean ± SEM. **p < 0.01 KO vs. WT; *n* = 4 for each group. Reprinted by permission from the publisher of Journal of Molecular Neuroscience, Nature/Springer/Palgrave; (Wang et al., 2015b).

**Figure 2:**
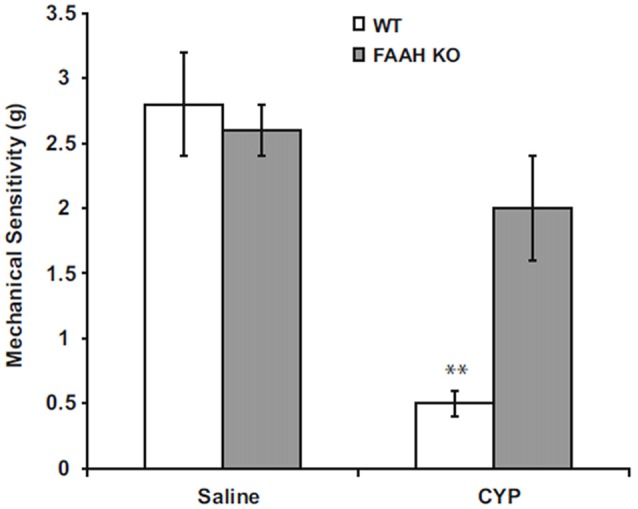
Peripheral mechanical sensitivity determined by application of von Frey monofilaments to hind paws 24 h after treatment with intraperitoneal saline (controls) or CYP (150 mg/kg). Mean ± SEM. **p < 0.01; vs. saline treated; *n* = 6–8. Reprinted by permission from the publisher of Journal of Molecular Neuroscience, Nature/Springer/Palgrave; (Wang et al., 2015b).

Suppression of FAAH activity by chemical inhibitors increases abundance of AEA in humans (Li et al., 2012) or mice (Ahn et al., 2011), and genetic deletion of functional FAAH in mice also increases concentrations of AEA (Cravatt et al., 2001; Lichtman et al., 2004). We observed increased AEA in bladders of FAAH-deficient (knock out or KO) mice, but abundance of 2-AG within the bladders of these mice was not affected in controls or by bladder inflammation (Wang et al., 2015b; Figure 1). Preclinical studies have demonstrated that increased AEA suppresses nociception in models of somatic and visceral pain when initiated prior to onset of pain (Cravatt et al., 2001; Lichtman et al., 2004; Merriam et al., 2011; Aizawa et al., 2016). We reported that severity of cyclophosphamide-induced bladder inflammation and associated referred pain was decreased in mice lacking FAAH (Wang et al., 2015b; Figure 2). We also found that systemic treatment of rats with established cystitis with a FAAH inhibitor given 1 h prior to testing diminished referred mechanical hypersensitivity (Merriam et al., 2011).

The analgesic effects of increased systemic AEA may be due to actions within the central nervous system (CNS) or peripheral tissues. Intravenous treatment of rats with URB937, a FAAH inhibitor with minimal penetration of the CNS decreased activity of bladder-specific afferent nerve in response to controlled filling of the bladder (Aizawa et al., 2014), and similar results were obtained by administration of URB937 prior to induction of cystitis by intravesical instillation of prostaglandin E_2_ (Aizawa et al., 2016).

AEA has been shown to be an agonist of the TRPV1 pro-nociceptive channel (Tognetto et al., 2001). The degree to which AEA activates TRPV1 varies among tissues and models of pain, but this effect has decreased enthusiasm for analgesic strategies entailing increased abundance of AEA alone. Simultaneous administration of inhibitors of FAAH and TRPV1, or compounds that act as antagonists against both, has produced promising, but mixed, results (Costa et al., 2010; de Novellis et al., 2011; Bashashati et al., 2017). This may be due in part to the fact that the relationship between CB1 and TRPV1 is far more complex than previously appreciated, and this interaction may be critically altered by tissue-specific metabolic factors (Fioravanti et al., 2008; Kim et al., 2008; De Petrocellis and Di Marzo, 2009).

Clinical trials of FAAH inhibitors failed to alleviate pain associated with osteoarthritis (Huggins et al., 2012). Of greater concern is a recent report that described serious adverse effects in a phase 1 trial of a FAAH inhibitor in humans, including coma and death in one subject and hospitalization of five other subjects, two with serious neurological symptoms (Mallet et al., 2016).

## Inhibition of Monoacylglycerol Lipase (MAGL)

Although 2-AG is far more abundant than AEA, less work has been done investigating the therapeutic potential of inhibition of MAGL to ameliorate visceral pain. In a mouse chronic neurogenic pain model, MAGL inhibition alleviated neuropathic pain (Kinsey et al., 2009; Ignatowska-Jankowska et al., 2015), and MAGL inhibition displayed reduced cannabimimetic effects compared to the CB1 receptor agonists (Ignatowska-Jankowska et al., 2015). Interestingly, combining COX and MAGL inhibition has shown a promising result of reducing neuropathic pain with minimal side effects (Crowe et al., 2015). MAGL inhibition increased paw skin 2-AG content and suppressed pain and inflammation subsequent to injection of formalin in the rat paw (Guindon et al., 2011; Ghosh et al., 2013). These data have clearly demonstrated that 2-AG is capable of inhibiting neuropathic and inflammatory pain.

A major obstacle to management of pain by inhibiting MAGL is the observation that increased systemic 2-AG actually results in enhanced response to painful stimuli due to desensitization of CB1 (Schlosburg et al., 2010; Kinsey et al., 2013; Ignatowska-Jankowska et al., 2014). CB1 receptors play a key role in the analgesic effects of endocannabinoids (Wilson and Nicoll, 2002; Agarwal et al., 2007; Lau et al., 2014), and chronic exposure to high concentrations of 2-AG results in CB1 desensitization and loss of CB1 analgesic input (Chanda et al., 2010; Lichtman et al., 2010; Ignatowska-Jankowska et al., 2014). There is differential expression of 2-AG among various organs, and it is possible that the degree of CB1 desensitization is proportionate to 2-AG concentrations, being most profoundly apparent in the brain (Lichtman et al., 2010). The observation of lower concentrations of 2-AG in peripheral structures is intriguing, because this raises the possibility of tissue-specific regulation of MAGL function to achieve local increases in 2-AG without inducing CB1 desensitization. Further, it has been reported that the anti-nociceptive and anti-inflammatory effects of chemical inhibitors of MAGL are preserved when these compounds are given chronically at low doses (Schlosburg et al., 2010; Ignatowska-Jankowska et al., 2014) in the absence of CB1 desensitization (Ghosh et al., 2013).

## Endocannabinoids and Inflammation

Endocannabinoids have been demonstrated to suppress inflammation in bladder (Wang et al., 2015b), joints (Barrie and Manolios, 2017), gut (Lee et al., 2016), skin (Lichtman et al., 2004) and CNS (Krishnan and Chatterjee, 2014). Our laboratory (Merriam et al., 2011) and others (Dinis et al., 2004) have reported increased AEA in inflamed bladders of rats, but we did not observe this in bladder inflammation in wild-type (WT) or FAAH KO mice (Figure 1; Wang et al., 2015b), and whether or not inflammation increases local synthesis of endocannabinoids remains unclear.

**Figure 3:**
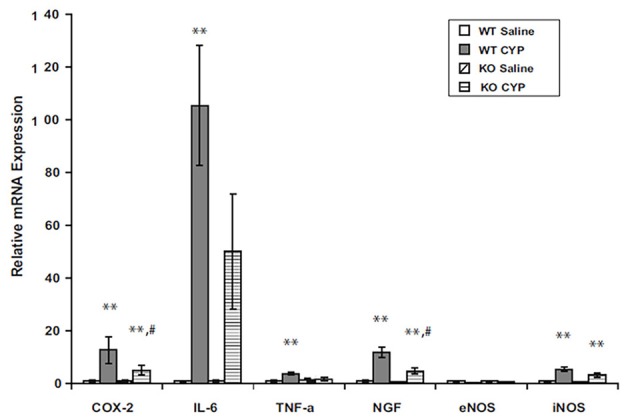
Bladder content of mRNA for inflammatory mediators in male WT and FAAH KO mice treated with saline (controls) or CYP (150 mg/kg) given intraperitoneally 3 h prior to sacrifice. Total bladder RNA was extracted, RT-PCR was performed, and results were normalized to signal for L19, a ribosomal protein. **p < 0.05 CYP vs. saline for each genotype; ^#^*p* < 0.05 CYP treated KO vs. CYP treated WT; *n* = 6–8 for each genotype and treatment. Reprinted by permission from the publisher of Journal of Molecular Neuroscience, Nature/Springer/Palgrave; (Wang et al., 2015b).

Mechanisms of suppression of inflammation by endocannabinoids are not completely understood, but AEA has been shown to inhibit mitogen-induced T- and B-cell lymphocyte proliferation by increasing apoptosis (Schwarz et al., 1994). AEA inhibited lipopolysaccharide (LPS)-mediated activation of NFκB (Nakajima et al., 2006) and has also been shown to block TNFα-induced activation of NFκB by suppression of I-κB kinase, the enzyme that mediates NFκB activation (Sancho et al., 2003). We observed decreased inflammation in bladders of FAAH-deficient (knock out or KO) mice with cyclophosphamide-induced cystitis (Wang et al., 2015b). Message COX2 and NGF were decreased in inflamed bladders of FAAH KO mice relative to those of wild type mice (Figure 3).

Binding of 2-AG to CB1 inhibits cyclooxygenase-2 in nerves resulting in suppression of MAPK/NFκB signaling (Zhang and Chen, 2008). 2-AG also prevented *in vitro* damage to hippocampal slices exposed to β-amyloid by a process mediated by CB1 binding that resulted in diminished ERK 1/2 phosphorylation, decreased NFκB activation, and reduced COX-2 expression (Chen et al., 2011). It has also been reported that AEA has the capacity to diminish Th-17 cell-mediated delayed-type hypersensitivity through increased IL-10 synthesis and subsequent microRNA production (Jackson et al., 2014). As research in this area continues, it is highly probable that other pathways by which endocannabinoids suppress inflammation will be discovered.

Inflammation plays a key role in release of substances that modulate nociception. Thus, it is highly likely that the analgesic effects of endocannabinoids may in part be due to their anti-inflammatory effects.

## Endocannabinoids and Bladder Pain

Evaluation of bladder pain in rodent models can be difficult, and the presence of bladder pain is most often inferred by evaluating referred mechanical sensitivity of the hind paws or abdominal wall or activity of bladder afferent nerves (Sadler et al., 2013; Stemler et al., 2013; Aizawa et al., 2014, 2016; Wang et al., 2014, 2015a,b; Girard et al., 2016). Inhibition of FAAH to increase AEA by systemic treatment with FAAH inhibitors (Merriam et al., 2011; Aizawa et al., 2014, 2016) or genetic disruption of function FAAH (Wang et al., 2015b) has been shown to suppress surrogates measurements of pain associated with bladder inflammation in rodents. However, it is unclear whether or not strategies applied systemically would be effective if adapted to tissue-specific (e.g., bladder) application.

Bladders and associated afferent nerves were isolated from mice treated *in vivo* with cyclophosphamide to induce inflammatory cystitis (Walczak et al., 2009; Walczak and Cervero, 2011). Afferent nerve activity was recorded during increasing intravesical pressure in inflamed bladders *ex vivo* in the presence or absence of intravesical cannabinoid agonists, and it was determined that intravesical cannabinoids suppressed afferent nerve fiber firing in inflamed bladders via CB1 activation (Walczak et al., 2009; Walczak and Cervero, 2011). We have also observed that intravesical administration of a CB1 agonist inhibited bladder responses to subsequent instillation of NGF (Wang et al., 2015a). These studies support the concept that intravesical manipulation of the endocannabinoid system may have the capacity to alter nociceptive signaling.

## Conclusion

Manipulation of the endocannabinoid system has emerged as an appealing alternative to opioids for management of severe bladder pain. However, the potential for undesirable side effects or lack of efficacy remain significant obstacles to advancement of this therapy. Emergence of dual inhibitors of endocannabinoid degradation and either activation of TRPV1 or COX may address many of the limitations of this approach. Information on the activity of endocannabinoids synthesized in peripheral tissues remains extremely limited. It remains unclear whether or not strategies to address organ-specific pain by manipulation of endocannabinoids is a viable option, but this is an intriguing alternative that has the potential to provide effective analgesia with minimal systemic side effects.

## Ethics Statement

All studies were approved by the University of Wisconsin Animal Care and Use Committee prior to performance. Procedures were consistent with guidelines provided by the Guide for the Care and Use of Laboratory Animals, 8th Edition, The National Academies Press, Washington, DC, USA.

## Author Contributions

DB and ZW participated in the concept experimental design, analysis and writing. ZW was directly involved in performing experiments.

## Conflict of Interest Statement

The authors declare that the research was conducted in the absence of any commercial or financial relationships that could be construed as a potential conflict of interest.
